# Immune Escape Adaptive Mutations in the H7N9 Avian Influenza Hemagglutinin Protein Increase Virus Replication Fitness and Decrease Pandemic Potential

**DOI:** 10.1128/JVI.00216-20

**Published:** 2020-09-15

**Authors:** Pengxiang Chang, Joshua E. Sealy, Jean-Remy Sadeyen, Sushant Bhat, Deimante Lukosaityte, Yipeng Sun, Munir Iqbal

**Affiliations:** a The Pirbright Institute, Pirbright, United Kingdom; b College of Veterinary Medicine, China Agricultural University, Beijing, China; University of North Carolina at Chapel Hill

**Keywords:** H7N9, avian viruses, immune escape, influenza, poultry, replication fitness, zoonotic infections

## Abstract

H7N9 avian influenza viruses (AIVs) continue to evolve and remain a huge threat to human health and the poultry industry. Previously, serially passaging the H7N9 A/Anhui/1/2013 virus in the presence of homologous ferret antiserum resulted in immune escape viruses containing amino acid substitutions alanine to threonine at residues 125 (A125T) and 151 (A151T) and leucine to glutamine at residue 217 (L217Q) in the hemagglutinin (HA) protein. These HA mutations have also been found in field isolates in 2019. To investigate the potential threat of serum escape mutant viruses to humans and poultry, the impact of these HA substitutions, either individually or in combination, on receptor binding, pH of fusion, thermal stability, and virus replication were investigated. Our results showed the serum escape mutant formed large plaques in Madin-Darby canine kidney (MDCK) cells and grew robustly *in vitro* and *in ovo*. They had a lower pH of fusion and increased thermal stability. Of note, the serum escape mutant completely lost the ability to bind to human-like receptor analogues. Further analysis revealed that N-linked glycosylation, as a result of A125T or A151T substitutions in HA, resulted in reduced receptor-binding avidity toward both human and avian-like receptor analogues, and the A125T+A151T mutations completely abolished human-like receptor binding. The L217Q mutation enhanced the H7N9 acid and thermal stability while the A151T mutation dramatically decreased H7N9 HA thermal stability. To conclude, H7N9 AIVs that contain A125T+A151T+L217Q mutations in the HA protein may pose a reduced pandemic risk but remain a heightened threat for poultry.

**IMPORTANCE** Avian influenza H7N9 viruses have been causing disease outbreaks in poultry and humans. We previously determined that propagation of H7N9 virus in virus-specific antiserum gives rise to mutant viruses carrying mutations A125T+A151T+L217Q in their hemagglutinin protein, enabling the virus to overcome vaccine-induced immunity. As predicted, these immune escape mutations were also observed in the field viruses that likely emerged in the immunized or naturally exposed birds. This study demonstrates that the immune escape mutants also (i) gained greater replication ability in cultured cells and in chicken embryos as well as (ii) increased acid and thermal stability but (iii) lost preferences for binding to human-type receptor while maintaining binding for the avian-like receptor. Therefore, they potentially pose reduced pandemic risk. However, the emergent virus variants containing the indicated mutations remain a significant risk to poultry due to antigenic drift and improved fitness for poultry.

## Abstract (precis)

Avian influenza H7N9 viruses have been causing disease outbreaks in poultry and humans. We previously determined that propagation of H7N9 virus in virus-specific antiserum gives rise to mutant viruses carrying mutations A125T+A151T+L217Q in their hemagglutinin protein, enabling the virus to overcome vaccine-induced immunity. As predicted, these immune escape mutations were also observed in the field viruses that likely emerged in the immunized or naturally exposed birds. This study demonstrates that the immune escape mutants also (i) gained greater replication ability in cultured cells and in chicken embryos as well as (ii) increased acid and thermal stability but (iii) lost preferences for binding to human-type receptor while maintaining binding for the avian-like receptor. Therefore, they potentially pose reduced pandemic risk. However, the emergent virus variants containing the indicated mutations remain a significant risk to poultry due to antigenic drift and improved fitness for poultry.

## INTRODUCTION

A novel H7N9 low-pathogenicity avian influenza (LPAI) virus strain emerged in birds in China. This virus showed the ability to cause severe to fatal disease in humans through zoonotic transmission from infected birds, and the first human infection with H7N9 was reported in China in February 2013. Since then, there have been 1,568 confirmed human infections with an ∼40% case fatality rate (1). The continued circulation of LPAI virus in poultry in China led to evolutionary changes in the virus genes including insertion of polybasic amino acid residues at the hemagglutinin (HA) cleavage site. This evolutionary change alters the LPAI virus phenotype to a high-pathogenicity avian influenza (HPAI) virus phenotype, increasing virus ability to cause up to 100% mortality in infected chickens (2). Evidence also suggests that compared to the LPAI virus, the HPAI H7N9 phenotype can cause more severe clinical disease in ducks and mice as well as in ferrets (3, 4). However, no increased pathogenicity of the virus within human cases has been detected related to the HPAI A(H7N9) virus (5). Serological studies have indicated that the HPAI H7N9 virus is antigenically distinct from the LPAI H7N9 virus, which prompted the World Health Organization (WHO) to update its recommendation for the H7N9 candidate vaccine virus composition to include the HPAI H7N9 viruses (A/Guangdong/17SF003/2016 [H7N9]) (6, 7). Further research revealed that an amino acid substitution at residue 217 from leucine to glutamine (L217Q) (mature H7 numbering used throughout; 217 is equivalent to 226 in H3 numbering) is responsible for significant antigenic differences between low- and high-pathogenicity H7N9 viruses (7).

Given the risk of H7N9 to humans and to the poultry industry, the Chinese government implemented a mass vaccination program against H7N9 in poultry in 2017. The chosen HA antigen was derived from an A/Anhui/1/2013 (H7N9) (referred to as Anhui/13)-like virus. Since its use, the number of poultry outbreaks along with human infections has dropped dramatically, with only three human infection cases reported in epidemic wave six during 2016 to 2017 and one human infection case reported in epidemic wave seven during 2017 to 2018 (1). Despite the reduction in disease outbreaks via vaccination, these viruses have not been eradicated, with continued sporadic isolation of LPAI and HPAI H7N9 viruses posing a threat to poultry and human health (4). The continued circulation of the virus in birds carrying virus-neutralizing antibodies induced through vaccination or natural exposure drives viruses to evolve and escape from the immune pressure. To imitate this natural antigenic drift scenario *in vitro*, propagation of influenza viruses in cultured cells in the presence of a subneutralizing concentration of virus-specific polyclonal antiserum has been performed to assess the antigenic evolution of HPAI H5N1 and seasonal influenza viruses (8, 9). In our previous study, serially passaging the prototype LPAI H7N9 virus (A/Anhui/1/2013) in the presence of homologous ferret antiserum resulted in the emergence of immune escape variants containing amino acid substitutions alanine to threonine at residue 125 (A125T), alanine to threonine at residue 151 (A151T), and L217Q in HA (these mutations correspond to A135T, A160T, and L226Q in H3 numbering, respectively) (7). Hemagglutination inhibition (HI) assays showed that the L217Q mutation alone was mainly responsible for the antigenic change. Incorporation of the additional mutation A125T did not dramatically affect the HI titers observed with the L217Q mutation alone. However, the additional A151T mutation lessened the antigenic change led by the L217Q substitution (7). These identified adaptive mutations (A125T+A151T+L217Q) using *in vitro* assays have also been observed in field isolates infecting poultry and humans in 2019 (10). It is, therefore, more likely that the emergent variant viruses can escape from immunity induced by the widely used H7N9 vaccine. The mutation at amino acid residue 217 is known to modulate influenza virus host range, and substitutions at amino acid residues 125 and 151 target virus HA protein glycosylation status. Therefore, it could be predicted that the evolutionary changes at these key amino acid residues could also influence virus pathobiological properties. Studies investigating transmission of H5N1 avian influenza viruses (AIVs) in ferrets revealed that efficient human-to-human transmission requires a reduced threshold for pH, increased HA thermal stability, and the shift of the HA receptor-binding preferences from avian to human (11, 12). The H7N9 viruses isolated from humans showed comparable binding preferences for both human-like receptor analogue α-2,6-sialyllactosamine (6SLN) and avian-like receptor analogue α-2,3-sialyllactosamine (3SLN) (13). The L217Q substitution in HA has been linked to the switch of avian receptors to human receptors in H2, H3, and H4 AIVs (14–16). However, LPAI H7N9 virus (Anhui/13) maintains its dual receptor-binding capacity despite the L217Q substitution (17). Instead, three mutations lysine (K)193T, valine (V)186K, and glycine (G)228 serine (S) were shown to switch the H7N9 receptor binding to human-like receptor specificity (18). In terms of HA thermal stability, the H7N9 AIVs appeared progressively less heat labile over time. Recent LPAI and HPAI H7N9 virus phenotypes have been shown to be even more stable than the H1N1 pandemic virus (2). In general, avian influenza viruses, such as H5N1, H7N7, and H9N2, prefer higher pH (5.6 to 6.0) for cellular fusion into avian hosts. In comparison, the human-adapted seasonal influenza viruses require relatively lower pH (5.0 to 5.4) for efficient fusion and entry into human cells (19). The LPAI H7N9 viruses have a pH of fusion of around 5.6 to 5.8. However, the HPAI H7N9 virus appears to fuse at pH 5.4 or lower; a recent study showed that the glutamate (E)64K substitution in HA2 was responsible for the difference of pH of fusion between LPAI and HPAI H7N9 viruses (20, 21).

In this study, we systematically investigated the impacts of the emergent immune escape variants carrying substitutions A125T, A151T, and L217Q individually or in combination on the virus infectivity, receptor binding, pH of fusion, and HA thermal stability. The results define the potential risks from the contemporary field H7N9 AIV with those HA mutations to human and animal health.

## RESULTS

### N-linked glycosylation of amino acid residue 123 and 149 as a result of A125T and A151T mutations in H7N9 HA.

**FIG 1:**
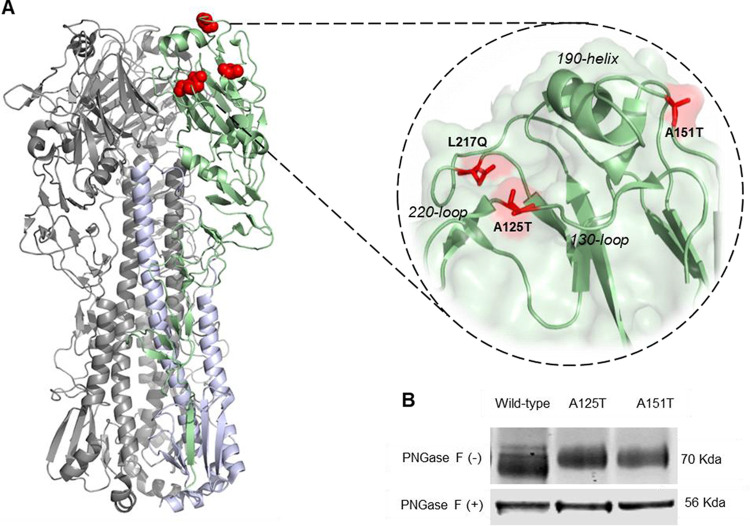
N-linked glycosylation at amino acid residues 123 and 149 as a result of A125T and A151T substitutions in the HA of H7N9 avian influenza virus. (A) Location of amino acid residue A125T, A151T, and L217Q substitutions identified in the HA of A/Anhui/1/2013 virus propagated in subneutralizing concentrations of homologous ferret antiserum. This image was generated from a HA trimer three-dimensional structure of H7N9 influenza virus (A/Shanghai/1/2013) (Protein Data Bank [PDB] accession number 4LN3). (B) SDS-PAGE analysis of N-linked glycosylation at amino acid residues 123 and 149 as a result of A125T and A151T substitutions in the HA of H7N9 avian influenza virus. Purified recombinant Anhui/13 wild-type virus or its variants with A125T or A151T mutation in HA were treated (+) or not (−) with PNGase F and were then lysed and subjected to SDS-PAGE analysis.

We previously showed that serially passaging the LPAI H7N9 avian influenza virus A/Anhui/1/2013 in the presence of homologous ferret antiserum selected the immune escape variants containing A125T+A151T+L217Q in the HA protein (7). Structurally, the amino acid residues 125 and 217 are both located in the vicinity of the receptor-binding site while amino acid residue 151 is positioned at the distal tip of HA (Fig. 1A). The substitutions at these residues, A125T and A151T, introduce N-linked glycosylation motifs (N-X-T, where X is any amino acid other than proline). To confirm the glycosylation pattern at these sites, we generated recombinant mutant viruses via reverse genetics (RG) containing HA and neuraminidase (NA) from Anhui/13 and six internal segments from PR8 virus (A/Puerto Rico/8/34 [H1N1]). The mutations A125T and A151T in HA were generated via site-directed mutagenesis with primers described in a previous study (7). The SDS-PAGE analysis showed that mutant RG viruses with HA containing an A125T or A151T mutation had a higher molecular weight than the wild-type-like RG virus carrying A125 and A151 residues (Fig. 1B). After deglycosylation by peptide-N-glycosidase F (PNGase F) enzyme, all samples including the wild type, the A125T mutant, and the A151T mutant showed equal electrophoretic mobility. These results confirmed the addition of glycan to the asparagine at amino acid residue 123 and 149 when substitutions A125T and A151T, respectively, are present in H7N9 HA.

### Serum escape mutant formed larger plaques and replicated robustly *in vitro* and *in ovo*.

**FIG 2:**
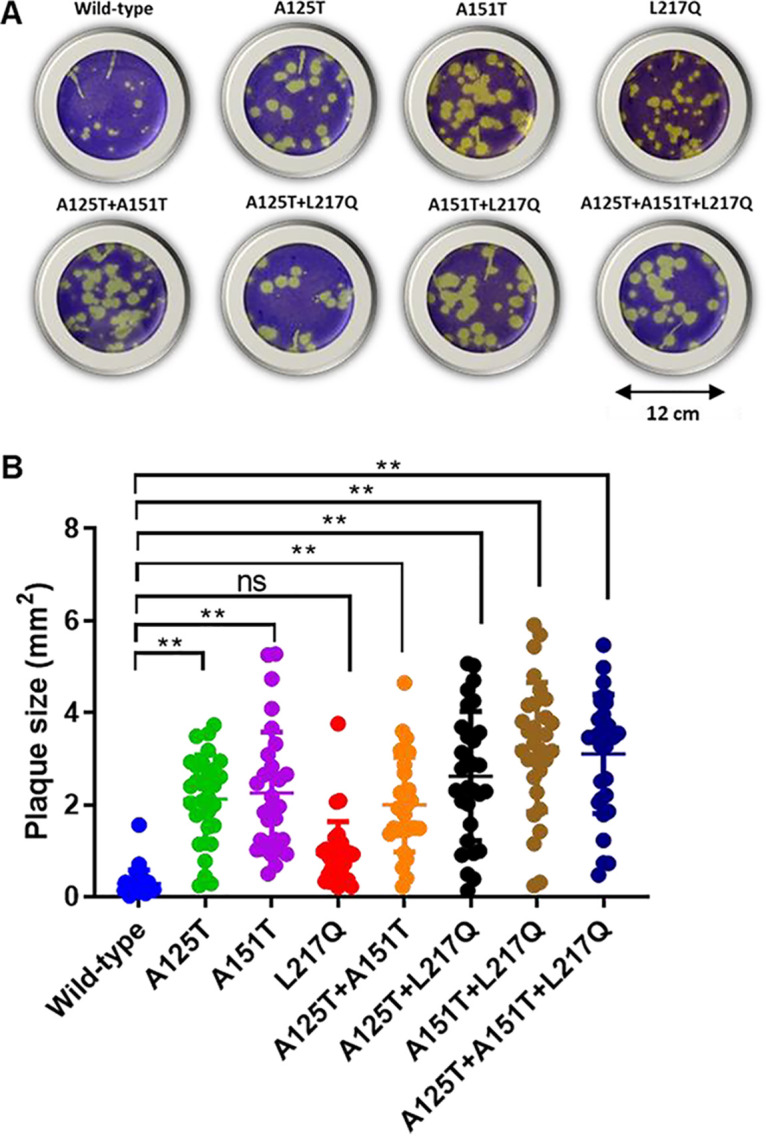
Plaque morphology of recombinant Anhui/13 virus and its variants in MDCK cells. (A) The plaque assay was performed in MDCK cells with recombinant Anhui/13 virus (referred to as the wild-type virus). The HA and NA from Anhui/13 virus and the internal segments from PR8 virus or its mutants with indicated amino acid substitutions. (B) The plaque size for the recombinant Anhui/13 wild-type virus and its variants in MDCK cells. Thirty plaques were randomly selected for each virus to measure the plaque diameter. Error bar = standard error of mean. *, *P* < 0.05; **, *P* < 0.001; ns = not significant. Results shown are representative of at least three experimental repeats.

**FIG 3:**
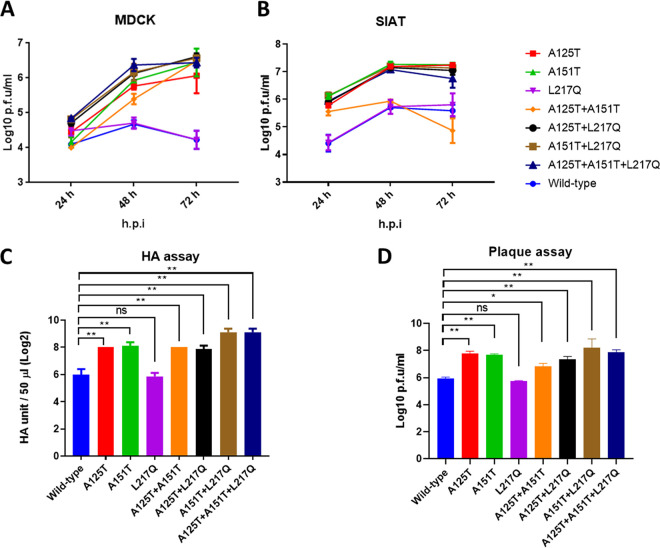
Replication of recombinant Anhui/13 and its variants *in ovo*, MDCK, and SIAT cells. The replication kinetic of Anhui/13 wild-type virus and its mutants in MDCK (A) and SIAT (B) cells (MDCK cells modified to express a higher density of α-2,6 human receptors). Cells were infected with a multiplicity of infection (MOI) of 0.001, supernatants were collected at the indicated time points, and the virus titers were determined by plaque assays in MDCK cells. The 10-day-old embryonated eggs were inoculated with 100 PFU of Anhui/13 wild-type virus and its mutants for 48 h before allantoic fluid was harvested and the viral replication was determined by HA assay (C) and plaque assay (D). Error bar = standard error of mean. *, *P* < 0.05; **, *P* < 0.001; ns = not significant. h.p.i. = hour postinfection.

To evaluate the effects of immune escape mutations in the HA on virus infectivity and replication fitness, we generated a series of reverse genetics-based mutant viruses carrying the indicated mutations A125T, A151T, and L217Q individually or in all possible combinations in the HA (A125T, A151T, L217Q, A125T+A151T, A125T+L217Q, A151T+L217Q, and A125T+A151T+L217Q) by site-directed mutagenesis. Like the Anhui/13 wild-type virus, the L217Q mutant formed relatively small plaques. However, all of the other mutant viruses formed significantly larger plaques than the wild-type viruses (Fig. 2A and B). To further assess the effects of those mutations on virus replication, we assessed the propagation for each of the viruses in mammalian Madin-Darby canine kidney (MDCK) cells and MDCK-SIAT1 (SIAT) cells, which have been modified to express a higher density of α-2,6 human receptors (22). Consistent with the smaller plaques formed by wild-type virus and the L217Q mutant, these two viruses replicated poorly in MDCK and SIAT cells (Fig. 3A). At 24 h postinfection, only the A125T+L217Q, A151T+L217Q, and the serum escape mutant replicated to significantly higher titers than the Anhui/13 wild-type virus in MDCK cells (Table 1). However, with the exceptions of the L217Q virus, all of the other mutant viruses replicated to a significantly higher titer than the wild-type virus at 48 and 72 h postinfection in MDCK cells (Fig. 3A; Table 1). Apart from the L217Q and A125T+A151T mutants, all of the other mutants replicated to a significantly higher titer than the Anhui/13 wild-type virus at all time points postinfection in SIAT cells (Fig. 3B; Table 2). Interestingly, the A125T+A151T mutant replicated to a significantly higher titer than the wild-type virus at 24 h postinfection, while the titers were comparable to those of the wild-type virus at 48 h and 72 h postinfection in SIAT cells. To further assess the effects of these mutations on virus replication, 10-day old embryonated eggs were inoculated with 100 PFU of Anhui/13 wild-type virus and its mutants for 48 h, and the virus titers were determined by HA assay (Fig. 3C) and plaque assay (Fig. 3D). The Anhui/13 wild-type virus and its L217Q mutant grew to only ∼64 HA units/50 μl. The HA titer of the serum escape mutant was comparable to that of the virus containing the A151T+L217Q double mutation, reaching ∼512 HA units/50 μl. The A125T, A151T, A125T+A151T, and A125T+L217Q mutant viruses replicated to ∼256 HA units/50 μl, which was significantly higher than the wild-type virus and the virus with the L217Q mutation alone. The plaque assay results showed a similar pattern as the HA assay, with the serum escape mutant and the A151T+L217Q mutant replicated to the highest titers (∼10^8^ PFU/ml), while the Anhui/13 wild-type virus and its L217Q mutant replicated to the lowest titers (∼10^6^ PFU/ml). To conclude, the serum escape mutants demonstrated enhanced replication both *in vitro* and *in ovo*, and the glycosylation at amino acid 123 or 149 contributed to virus replication fitness.

**TABLE 1:** The two-way ANOVA of the H7N9 virus replication kinetics in MDCK cells

Tukey's multiple-comparisons test parameters^*a*^	Significant	Adjusted *P* value
24 h Wild type vs 24 h A125T	No	0.8141
24 h Wild type vs 24 h A151T	No	>0.9999
24 h Wild type vs 24 h L217Q	No	0.6573
24 h Wild type vs 24 h A125T+A151T	No	>0.9999
24 h Wild type vs 24 h A125T+L217Q	Yes	0.037
24 h Wild type vs 24 h A151T+L217Q	Yes	0.0051
24 h Wild type vs 24 h A125T+A151T+L217Q	Yes	0.0031
48 h Wild type vs 48 h A125T	Yes	<0.0001
48 h Wild type vs 48 h A151T	Yes	<0.0001
48 h Wild type vs 48 h L217Q	No	>0.9999
48 h Wild type vs 48 h A125T+A151T	Yes	0.0047
48 h Wild type vs 48 h A125T+L217Q	Yes	<0.0001
48 h Wild type vs 48 h A151T+L217Q	Yes	<0.0001
48 h Wild type vs 48 h A125T+A151T+L217Q	Yes	<0.0001
72 h Wild type vs 72 h A125T	Yes	<0.0001
72 h Wild type vs 72 h A151T	Yes	<0.0001
72 h Wild type vs 72 h L217Q	No	>0.9999
72 h Wild type vs 72 h A125T+A151T	Yes	<0.0001
72 h Wild type vs 72 h A125T+L217Q	Yes	<0.0001
72 h Wild type vs 72 h A151T+L217Q	Yes	<0.0001
72 h Wild type vs 72 h A125T+A151T+L217Q	Yes	<0.0001

a Wild-type and mutant viruses with indicated amino acid substitutions were generated via reverse genetically contained HA and NA from Anhui/13 virus and the internal gene segments from PR8 virus. MDCK cells were infected with viruses at an MOI of 0.001.

**TABLE 2:** The two-way ANOVA of the H7N9 virus replication kinetics in SIAT cells

Tukey's multiple-comparisons test parameters^*a*^	Significant	Adjusted *P* value
24 h Wild type vs 24 h A125T	Yes	<0.0001
24 h Wild type vs 24 h A151T	Yes	<0.0001
24 h Wild type vs 24 h L217Q	No	>0.9999
24 h Wild type vs 24 h A125T+A151T	Yes	<0.0001
24 h Wild type vs 24 h A125T+L217Q	Yes	<0.0001
24 h Wild type vs 24 h A151T+L217Q	Yes	<0.0001
24 h Wild type vs 24 h A125T+A151T+L217Q	Yes	<0.0001
48 h Wild type vs 48 h A125T	Yes	<0.0001
48 h Wild type vs 48 h A151T	Yes	<0.0001
48 h Wild type vs 48 h L217Q	No	>0.9999
48 h Wild type vs 48 h A125T+A151T	No	0.9976
48 h Wild type vs 48 h A125T+L217Q	Yes	<0.0001
48 h Wild type vs 48 h A151T+L217Q	Yes	<0.0001
48 h Wild type vs 48 h A125T+A151T+L217Q	Yes	<0.0001
72 h Wild type vs 72 h A125T	Yes	<0.0001
72 h Wild type vs 72 h A151T	Yes	<0.0001
72 h Wild type vs 72 h L217Q	No	0.9993
72 h Wild type vs 72 h A125T+A151T	Yes	0.0071
72 h Wild type vs 72 h A125T+L217Q	Yes	<0.0001
72 h Wild type vs 72 h A151T+L217Q	Yes	<0.0001
72 h Wild type vs 72 h A125T+A151T+L217Q	Yes	<0.0001

a Wild-type and mutant viruses with indicated amino acid substitutions were generated via reverse genetically contained HA and NA from Anhui/13 virus and the internal gene segments from PR8 virus. SIAT cells were infected with viruses at an MOI of 0.001.

### Serum escape mutant completely lost human-like receptor binding.

**FIG 4:**
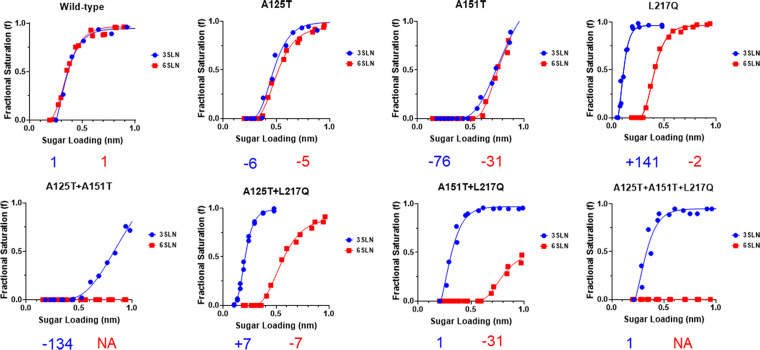
Receptor-binding profiles of recombinant Anhui/13 virus and its variants. The binding of purified recombinant Anhui/13 wild-type virus and its mutants with indicated HA substitutions to avian (α-2,3-SLN, shown in blue) and human (α-2,6-SLN, shown in red) receptor analogues by biolayer interferometry. The numbers below each figure show the fold change of receptor binding of indicated viruses to avian (α-2,3-SLN, shown in blue) and human (α-2,6-SLN, shown in red) receptor analogues compared to those of the recombinant Anhui/13 wild-type virus. −, reduction; +, increase; NA, not applicable. Data is the combination of two repeats for each virus and receptor analogue combination.

Influenza viruses adapted to infect humans preferentially bind to human-like receptors (α-2,6-sialic acid receptors), whereas avian influenza viruses adapted to infect birds preferentially bind to avian-like receptors (α-2,3-sialic acid receptors) (23). The receptor binding switch is one of the key determinants for the interspecies transmission of influenza viruses. It was reported previously that H7N9 viruses isolated from humans had strong binding avidity to both α-2,3- and α-2,6-linked sialic acid receptors (13). To examine the impacts of immune escape mutations on virus receptor-binding properties, we utilized biolayer interferometry to characterize the receptor-binding profiles of these viruses to the avian-like and human-like receptor analogues, 3SLN and 6SLN. In agreement with Xiong et al., the H7N9 Anhui/13 virus showed comparable binding to both 3SLN and 6SLN receptor analogues (Fig. 4) (13). Glycosylation at either amino acid position 123 (A125T) or 149 (A151T) of H7N9 HA resulted in reduced receptor binding toward both 3SLN and 6SLN analogues, and the reduction was more dramatic with the A151T mutant (∼76-fold reduction to 3SLN and ∼31-fold reduction to 6SLN) than with the A125T mutant (∼6-fold reduction to 3SLN and ∼5-fold reduction to 6SLN). Interestingly, virus with glycosylation at both residues 123 (A125T) and 149 (A151T) completely lost binding to the 6SLN receptor analogue while maintaining weak binding to the 3SLN receptor analogue. L217Q has been linked to the switch of avian receptors to human receptors in H2, H3, and H4 avian influenza viruses (14–16). However, here, we only saw a slight decrease (∼2-fold) of L217Q mutants to human-like receptor analogue 6SLN, whereas the binding toward avian-like receptor analogue 3SLN increased dramatically (∼141-fold) when compared with that of the wild-type virus. The increased 3SLN analogue binding by the L217Q mutation was also true in the background of A125T, A151T, or A125T+A151T mutations. The serum escape mutant (A125T+A151T+L217Q) showed comparable binding toward the 3SLN analogue with wild-type virus, whereas there was no detectable binding toward the 6SLN receptor analogue. Compared with the wild-type virus, the mutant containing A125T+L217Q substitutions showed reduced binding to the 6SLN analogue (∼7-fold) but greater binding to the 3SLN analogue (∼7-fold). The A151T+L217Q mutant showed weaker binding to the 6SLN analogue (∼31-fold) but maintained similar binding toward the 3SLN analogue. These data suggest that serum escape mutations A125T, A151T, or L217Q in the HA (individually or in combination) play a vital role in receptor-binding avidity of the H7N9 virus. In particular, the A125T+A151T+L217Q mutations completely abolished the human-like receptor binding while maintaining the avian-type receptor binding of H7N9 virus.

### Serum escape mutant eluted from chicken red blood cells at a faster rate.

**FIG 5:**
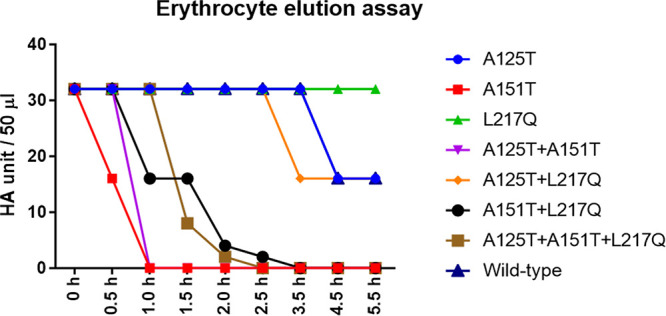
Erythrocyte elution assay of recombinant Anhui/13 virus and its variants. The 32 HA units of recombinant Anhui/13 wild-type virus and its mutants with indicated HA substitutions were 2-fold serially diluted and mixed with 1% chicken red blood cells. The HA titer was recorded over 5.5 h. Results shown are representative of three experimental repeats.

To evaluate the functional significance of the receptor-binding data by biolayer interferometry, we measured the elution of viruses from chicken red blood cells (RBCs) that possess both α-2,3- and α-2,6-linked sialic acid receptors (24). Consistent with the previous publication, which showed that the poor replication of the H7N9 AIV in MDCK cells was associated with strong HA receptor binding relative to weak NA cleavage of receptors (25), recombinant Anhui/13 wild-type virus bound tightly to RBCs as indicated by slow blood elution within 5.5 h (Fig. 5). The L217Q mutant virus, which had enhanced avidity for avian-like receptor analogue 3SLN and a slight decrease in avidity for human-like receptor analogue 6SLN, did not elute from RBCs within 5.5 h. In agreement with the biolayer interferometry results, all mutants containing A151T substitutions demonstrated faster elution from RBCs compared to that of the wild-type virus. The A151T and A151T+A125T mutants showed the quickest RBC elution; both eluted completely from RBCs within 1 h. In comparison with the A151T mutant, the A151T+L217Q mutant virus, which showed increased binding toward the 3SLN analogue, eluted slower from RBCs. The serum escape mutant that completely lost human-like receptor binding eluted slightly quicker than the A151T+L217Q mutant. Though glycosylation by A125T substitution also caused a reduction in receptor-binding affinity toward both α-2,3- and α-2,6-linked sialic acid receptors, the change appeared to be not enough to affect the virus elution from RBCs. However, in the background of the L217Q mutation, the additional A125T mutation resulted in a faster release of virus from the RBCs. Taken together, consistent with the biolayer interferometry data, glycosylation at residues 123 and 149 that decreased the receptor-binding avidity resulted in faster virus elution from RBCs.

### Serum escape mutant demonstrated reduced pH of fusion and increased HA thermal stability.

**FIG 6:**
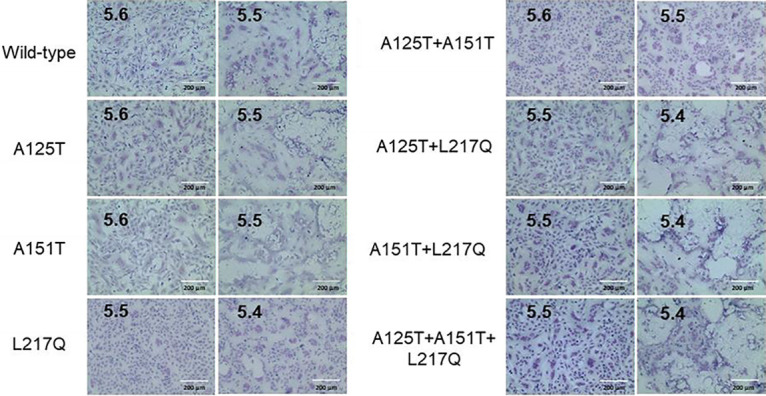
pH fusion threshold of recombinant Anhui/13 virus and its variants. Syncytium formation in Vero cells infected with recombinant Anhui/13 wild-type virus and its mutants with indicated HA substitutions. The pH at which 50% of maximum syncytium formation was taken as the predicted pH of fusion (shown on the left). The syncytium formation at 0.1 pH unit lower than the fusion threshold were shown on the right as controls. Results shown are representative of three experimental repeats.

The pH of fusion has been shown to play an important role in host adaptation and transmission. Consistent with the previous report, wild-type Anhui/13 triggered fusion at pH 5.6 or lower (26). The serum escape mutant containing the A125T+A151T+L217Q mutations in HA fused at pH 5.5 or lower (Fig. 6). Further analysis showed that the L217Q substitution in HA is responsible for the reduced fusion pH of the serum escape mutant. The glycosylation at residues 123 and 149 as a result of the A125T, A151T, or A125T+A151T mutation did not affect the pH fusion threshold.

**FIG 7:**
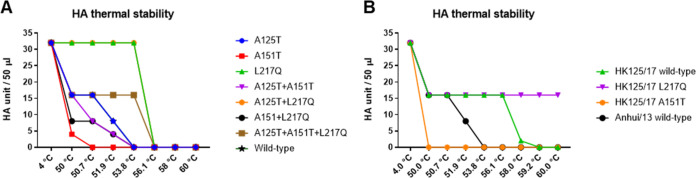
The impacts of A125T, A151T, and L217Q mutations on HA thermal stability. (A) HA thermal stability of recombinant Anhui/13 wild-type virus and its mutants with indicated HA substitutions. (B) HA thermal stability of recombinant HK125/17 virus and its mutants with A151T or L217Q substitutions in HA. The Anhui/13 wild-type virus served as control. The 32 HA units of recombinant viruses were either left at 4°C as a control or heated to 50°C, 50.7°C, 51.9°C, 53.8°C, 56.1°C, 58.0°C, 59.2°C, and 60°C for 30 min before the HA assay. Results shown are representative of three experimental repeats.

In addition to receptor binding and pH of fusion, HA stability also plays a vital role in AIV evolution (27). It has been a long-held notion that the influenza virus pH of fusion is inversely correlated to its thermostability (28–30). In agreement with those reports, L217Q mutation in HA resulted in a reduced pH of fusion threshold and elevated HA thermal stability (Fig. 7A). However, glycosylation at H7N9 HA residue 149 as a result of the A151T mutation did not affect the pH of fusion but dramatically decreased the thermal stability. To make sure this was not a strain-specific effect, we introduced those two mutations into the HA of the A/Hong Kong/125/2017 (HK125/17) virus, which is the human isolate from H7N9 epidemic wave five in 2016/2017. The recombinant HK125/17 wild-type virus showed higher thermal stability than the Anhui/13 wild-type virus, and the L217Q substitution in HA led to increased thermal stability (Fig. 7B). However, the A151T mutation dramatically destabilized the HK125/17 virus, making it less stable than the Anhui/13 wild-type virus. The serum escape mutant carries mutations that have opposing effects on the HA thermal stability, namely, the A151T mutation weakened whereas the L217Q mutation stabilized HA stability. However, the overall HA stability of the serum escape mutant increased when compared to that of the wild-type virus (Fig. 7A). Together, these results indicated that the serum escape mutant viruses had increased acid and thermal stability in comparison to that of the wild-type Anhui/13 virus, and the L217Q substitution in HA was accountable for this difference.

### Bioinformatic analysis of the mutations identified in the serum escape mutant.

Polymorphism analysis was performed on HA sequences retrieved from the GISAID (Global Initiative on Sharing All Influenza Data) database from 1 January 2013 to 1 September 2019 in China. All mutations, apart from A125T+A151T and A125T+L217Q, were found in naturally occurring H7N9 isolates (Table 3). The A151T+L217Q mutation emerged recently and was first detected in 2016/2017 and then persisted until 2018/2019, though the number of isolates was low. The mutations A125T+A151T+L217Q identified in the serum escape mutant were found in virus isolates from a recent fatal human case and from a local live bird market in 2019. The Chinese government implemented a mass vaccination program against H7N9 in poultry in 2017. Therefore, the emergence of H7N9 AIVs containing A125T+A151T+L217Q substitutions might be a result of antigenic drift under vaccine-induced pressure.

**TABLE 3:** Polymorphism analysis of HA residues 125, 151, and 217, individually or in combinations, from 1 January 2013 to 1 September 2019

Period	% (no.) of sequence with the indicated HA mutation(s)^*a*^						
	A125T	A151T	L217Q	A125T+A151T	A125T+L217Q	A151T+L217Q	A125+A151T+L217Q
1 Jan. 2013 to 1 Sept. 2013	0	0	13.5 (28)	0	0	0	0
1 Sept. 2013 to 1 Sept. 2014	0.9 (6)	0.6 (4)	1.6 (11)	0	0	0	0
1 Sept. 2014 to 1 Sept. 2015	0	0.4 (1)	3.9 (10)	0	0	0	0
1 Sept. 2015 to 1 Sept. 2016	0	0	1.2 (1)	0	0	0	0
1 Sept. 2016 to 1 Sept. 2017	0	1.2 (8)	14.1 (94)	0	0	0.15 (1)	0
1 Sept. 2017 to 1 Sept. 2018	0	0	73.7 (14)	0	0	5.3 (1)	0
1 Sept. 2018 to 1 Sept. 2019	0	0	0	0	0	18.8 (3)	68.8 (11)

a All available H7N9 virus sequences were retrieved from the Global Initiative on Sharing All Influenza Data (GISAID) from 1 January 2013 to 1 September 2019 in China (1 January 2013 to 1 September 2013 [sequence number = 208], 1 September 2013 to 1 September 2014 [sequence number = 700], 1 September 2014 to 1 September 2015 [sequence number = 258], 1 September 2015 to 1 September 2016 [sequence number = 85], 1 September 2016 to 1 September 2017 [sequence number = 666], 1 September 2017 to 1 September 2018 [sequence number = 19], and 1 September 2018 to 1 September 2019 [sequence number = 16]). The number in the bracket shows the number of sequences that contain the indicated mutations.

## DISCUSSION

In our previous study, serially passaging the H7N9 viruses (A/Anhui/1/2013) in the presence of homologous ferret antiserum resulted in the emergence of escape viruses containing mutations A125T+A151T+L217Q in the HA (7). To assess the risk of the serum escape H7N9 virus variants to both poultry and humans, we studied the impacts of A125T, A151T, and L217Q substitutions in the HA gene, either individually or in different combinations, on virus infectivity *in vitro* and *in ovo*, virus receptor-binding profile, pH fusion threshold, and HA thermal stability. The serum escape mutants containing A125T+A151T+L217Q substitutions replicated to significantly higher titers than the wild-type Anhui/13 virus both *in vitro* and *in ovo* at various time points. They completely lost human-like receptor binding while maintaining the avian-like receptor binding, fused at lower pH, and had higher HA thermal stability. Further analysis showed that A125T+A151T double mutations that resulted in N-linked glycosylation at amino acids 123 and 149 were responsible for the loss of human-like receptor binding. The L217Q substitution was responsible for the increased acid and thermal stability.

The amino acid 217 polymorphism appeared to play multiple important roles in H7N9 AIV evolution. The L217Q substitution in HA has been linked to the switch of avian receptors to human receptors in H2, H3, and H4 avian influenza viruses (14–16). However, LPAI H7N9 virus (Anhui/13) maintains its dual receptor-binding capacity for both avian and human hosts despite the L217Q substitution (17). Here, we showed that the L217Q substitution in H7N9 HA only caused a slight decrease to human-like receptor analogue 6SLN, but the binding toward avian-like receptor analogue 3SLN was dramatically increased. This agrees with the results of a study by Schrauwen et al., who showed that the L217Q mutant virus predominantly bound to avian receptors but maintained residual binding to human receptors (26). In addition to the role in receptor binding, the L217Q substitution in HA also increased the H7N9 AIV thermal and acid stability, which has been indicated to be important for the mammalian adaptation and aerosol transmission of H5N1 AIV in ferrets (11, 12). However, in ducks, the transmissibility of H5N1 AIV was increased by substitutions that slightly reduced the pH of fusion, leading to higher shedding and greater environmental persistence (31). Decreased pH of fusion was also found to be critical for airborne transmissibility of AIV H9N2 between chickens (32). The contradictory impacts of the acid and thermal stability on the host adaptation indicated subtype or strain-specific differences. However, given that more than 90% of HPAI H7N9 viruses contains Q217 and their wide geographical spread within China, we propose that the slight increase of acid and thermal stability of H7N9 AIVs might favor virus transmission in poultry due to greater environmental persistence (7). We previously showed that amino acid residue 217 in the HA is also a key mediator of avian influenza H7N9 virus antigenicity (7). This agrees with the studies that identified amino acid residue 217 as an important antigenic epitope for H7N9 AIV by human monoclonal antibodies (33, 34). It has been shown that the L217Q substitution in AIV H7N9 resulted in poor transmission between pigs (35). Similarly, Sun et al. reported that LPAI H7N9 virus containing L217 transmitted well between ferrets. However, the two HPAI H7N9 viruses that contain Q217 could not transmit between ferrets (20). Therefore, the H7N9 AIV that contains Q217 in HA posed less zoonotic and pandemic risk than the virus that contains L217 in HA, even though L217Q did not completely abolish human receptor binding. Given the important antigenic epitope of amino acid 217, we predict that immune pressure through mass vaccination of poultry might have driven an evolutionary change, L217Q, in the HA of H7N9 AIV, increasing adaptation to avian host, which in turn might contribute to the sharp decrease of human cases of H7N9 AIV infection after epidemic wave five (7).

It was suggested that influenza A virus HA glycosylation compensates for antibody escape fitness costs (36). Therefore, the glycosylation by A125T and A151T mutations was probably to compensate the reduced virus replication of the L217Q mutant, which was the major mediator for the H7N9 AIV antigenic drift. We cannot rule out that the occurrence of A125T and A151T was a direct result of antibody pressure because it is a regular strategy for influenza viruses to escape the neutralizing antibodies by attaching glycans, which can sterically block the binding of antibodies to multiple sites (34, 37). The N-linked glycosylation as a result of the A125T mutation in the HA of H7N9 AIV has been reported previously to block the neutralizing antibodies and enhance the infectivity of pseudoviruses expressing the H7N9 AIV HA (37). Likewise, A125T substitution in HA of HPAI H7N7 virus was linked to the increased replication efficiency in MDCK and A549 cells (38). It is noteworthy that the A125T substitution has also been found to emerge in the HA of an H7N9 AIV isolated from the contact ferrets during a transmission experiment (39). Here, we showed that the A125T substitution added glycan at amino acid residue 123, resulting in reduced receptor affinity to both avian-like receptor 3SLN and human-like receptor 6SLN analogues, though its effect on the RBC elution is not evident. Corresponding to the decreased receptor binding, the A125T and A125T+L217Q mutants replicated significantly better than Anhui/13 wild-type virus and the L217Q mutant, respectively. Previous reports showed that poor replication of the H7N9 AIV in MDCK cells was associated with strong HA receptor binding relative to weak neuraminidase (NA) cleavage of receptors (25). Thus, the improved replication of the H7N9 AIV with an A125T mutation might be due to reduced HA receptor binding that restored the functional balance between HA and NA. We, therefore, propose that the occurrence of the A125T mutant in contact ferrets during the transmission study might be linked to improved virus fitness rather than receptor switch to human-like receptor binding. Our results are consistent with Ohuchi et al., showing that A125T mutation resulted in reduced receptor binding, which in turn led to quick blood elution from H7N1 AIVs (40). However, it has been reported previously that A125T mutation led to increased avian receptor binding of H7N7 and H7N9 AIVs using glycan microarray (41, 42). This discrepancy might be due to the different sizes of the N-glycans of the AIV HA when expressed in insect cells and mammalian cells. We previously showed that the A125T+L217Q mutant was antigenically distant from Anhui/13 wild type. Here, we showed that it replicated efficiently *in vitro* and *in ovo* and was more acid and thermal stable than the wild-type virus. Furthermore, it has been reported that glycosylation at 123 as a result of A125T substitution enhances the transmission of HPAI H7N1 between turkeys and chickens (43). All of these results indicated that H7N9 AIV containing A125T+L217Q double mutations in HA might be more fit in poultry than the wild-type virus, in particular under vaccine-induced antibody pressure. Surprisingly, there has been no report of H7N9 AIVs that contain only A125T+L217Q double substitution so far (GISAID data, accessed on 8 January 2020). The only strains that contain A125T+L217Q substitution in HA come along with additional A151T mutations, which were found in the human and environmental samples in the most recent H7N9 human case in Gansu, China.

Although potential glycosylation at amino acid 149 as a result of A151T mutation has been indicated in many publications due to the presence of N-linked glycosylation motif N-X-T/S, here for the first time, we validate this glycosylation in the HA of H7N9 AIV by SDS-PAGE analysis (7, 44, 45). The amino acid 151 located at the tip of HA has been identified as an egg adaptation marker (44). It was also shown to help the H7N9 AIV to escape antibody binding (34). Of note, we identified that A151T substitution in HA also resulted in a dramatic decrease in HA thermal stability while not affecting its acid stability. The weak thermal stability has been linked to poor environmental presence and might not benefit virus transmission (46). This finding might explain the poor transmission of HPAI H7N1 with glycosylation at amino acid 149 between turkeys and chickens (43).

The serum escape mutant replicated efficiently *in vitro* and *in ovo*, demonstrated enhanced acid and thermal stability, and demonstrated a loss of human-like receptor binding, leaving capacity for avian-like receptor binding only. Therefore, H7N9 viruses that contain these mutations might adapt to poultry and pose reduced pandemic risk. However, the work done here is only based on the *in vitro* and *in ovo* data with recombinant viruses with six internal gene segments from PR8 influenza virus; it is, therefore, crucial to study the impact of these mutations to whole H7N9 viruses in animal models.

## MATERIALS AND METHODS

### Ethics statement.

All animal studies and procedures were carried out in strict accordance with the guidance and regulations of European and United Kingdom Home Office regulations under project license number P68D44CF4. As part of this process, the work has undergone scrutiny and approval by the Animal Welfare Ethical Review Board (AWERB) at the Pirbright Institute. All of the influenza virus-related work was risk assessed and carried out adopting biosafety level 2 containment code of practice approved by The Pirbright Institute Biological Agents and Genetic Modification Safety Committee and the UK Health and Safety Executive.

### Viruses and cells.

The recombinant H7N9 viruses containing HA and NA from LPAI H7N9 Anhui/13 virus or H7N9 HK125/17 virus and the internal gene segments from A/Puerto Rico/8/34 (H1N1) (PR8) virus were generated as previously described (7). The recombinant H7N9 AIV A125T, A151T, L217Q, A125T+A151T, A125T+L217Q, A151T+L217Q, and A125T+A151T+L217Q (referred to as serum escape mutant) mutants of Anhui/13 and L217Q and A151T mutants of HK125/17 were generated via site-directed mutagenesis with primers described in a previous study (7). Rescued viruses were propagated in 10-day-old embryonated chicken eggs, and virus stocks were kept at −80°C.

The Madin-Darby canine kidney (MDCK), MDCK-SIAT1 (SIAT), human embryonic kidney 293T (HEK 293T), and Vero cells (ATCC) were maintained with Dulbecco’s modified Eagle’s medium (DMEM) (Gibco), supplemented with 10% fetal calf serum (FCS) (Gibco), 100 U/ml penicillin, and 100 μg/ml streptomycin (Gibco) at 37°C under a 5% CO_2_ atmosphere.

### Replication kinetics in MDCK cells and plaque assay.

MDCK cells were infected with H7N9 AIV at a multiplicity of infection (MOI) of 0.001 and incubated at 37°C for 1 h. The cells were then washed once with phosphate-buffered saline (PBS) and replenished with DMEM containing 2 μg/ml tosylsulfonyl phenylalanyl chloromethyl ketone (TPCK)-treated trypsin. The supernatants were taken at 24, 48, and 72 h postinfection and stored in −80°C before titration by plaque assay in MDCK cells. The plaque assay was carried out as previously described (47), and the MDCK cells were infected with H7N9 AIV for 1 h. The cells were then washed once with PBS before addition of minimum essential medium (MEM)-agarose overlay. The MEM-agarose overlay medium contains 0.6% agarose (Thermo Fisher), 1× MEM, 100 U/ml penicillin, 100 μg/ml streptomycin, 2 mM l-glutamine, 0.3% bovine serum albumin (BSA), 15 mM HEPES, 0.22% sodium bicarbonate, and 0.01% DEAE-dextran (all Sigma-Aldrich). The cells were further incubated at 37°C for 72 h before being fixed with 1% crystal violet (Sigma-Aldrich) in 20% methanol. The plaque images were taken by Panasonic camera FZ38, and 30 plaques were randomly selected for each virus to measure the plaque diameter.

### *In ovo* growth.

Ten-day-old embryonated chicken eggs were infected with 100 PFU of wild-type Anhui/13 virus or its variants. The eggs were incubated at 35°C for 48 h before allantoic fluid was harvested and titrated by hemagglutination (HA) assay.

### HA assay.

The HA titration assays were performed using 1% chicken red blood cells (RBC) as described in the WHO animal influenza training manual (48). Briefly, 50 μl of virus was added to the 96-well V-bottom plates (Greiner) and was then 2-fold serially diluted, followed by addition of 50 μl of 1% washed chicken RBCs. The plates were incubated for 30 min at room temperature before the HA titer was recorded until the highest dilution giving complete agglutination and presented as HA units/50 μl.

### Erythrocyte elution assay.

Recombinant H7N9 virus containing 32 HA units in a 50-μl volume was serially 2-fold diluted with PBS in 96-well V-bottom microtiter plates (Greiner). Each virus dilution was mixed with 50 μl of 1% chicken RBC. The plates were initially kept at 4°C for 1 h and then incubated at 37°C. The HA titer was recorded over 5.5 h.

### HA thermal stability assay.

The recombinant H7N9 avian influenza viruses were diluted with allantoic fluid harvested from noninfected embryonated chicken eggs to 32 HA units/50 μl. The viruses were then left at 4°C as control or heated at 50°C, 50.7°C, 51.9°C, 53.8°C, 56.1°C, 58.0°C, 59.2°C, and 60°C for 30 min before HA assay.

### Syncytium formation assays.

The pH of fusion for H7N9 AIV was determined by syncytium formation assays as previously described with slight modification (49). To determine the optimal number of viruses to use, the H7N9 AIVs were 2-fold serially diluted in DMEM to infect the Vero cells in 96-well plates for 1 h. The inoculum was then removed and washed with PBS before addition of growth medium for 15 h. The cells were then fixed with a methanol and acetone (1:1) mixture and then immunostained with anti-nucleoprotein (anti-NP) mouse monoclonal antibody followed by horseradish peroxidase-labeled rabbit anti-mouse immunoglobulins (Dako) as previously described (50). The virus concentration at the highest dilution still infecting 100% of the Vero cells was used to infect Vero cells in 96-well plates for the syncytium formation assays. At 16 h postinfection, cells were treated with 3 μg/ml TPCK-treated trypsin for 15 min and then exposed to PBS buffers with pH values ranging from 5.2 to 6.0 (at 0.1 unit increments) for 5 min. The PBS buffer was then replaced with DMEM with 10% FCS. The cells were further incubated for 3 h at 37°C to allow for syncytium formation before being fixed with methanol and acetone (1:1) mixture and stained with Giemsa stain (Sigma-Aldrich) for 3 h at room temperature. The pH at which 50% of maximum syncytium formation estimated was taken as the predicted pH of fusion. Images were taken on the Evos XL cell imaging system (Life Technologies).

### Virus purification and biolayer interferometry.

Viruses were purified as previously described (28). Briefly, virus harvested from egg allantoic fluid was pelleted by ultracentrifugation and then purified through a continuous 30% to 60% (wt/vol) sucrose gradient. The virus concentration was determined using an enzyme-linked immunosorbent assay against the virus NP as described previously (51). Virus binding to receptor analogues was measured on an Octet Red instrument (ForteBio). The biotinylated α-2,3- and α-2,6-linked sialyllactosamine sugars (3SLN and 6SLN, respectively) were purchased from GlycoNZ. Virus was diluted in HBS-EP buffer (Teknova) containing 10 μM oseltamivir carboxylate (Roche) and 10 μM zanamivir (GSK) to a concentration of 100 pM. Equilibrium responses for virus binding were plotted as a function of the amount of sugar immobilized on the biosensor calculated from the response during the sugar loading step (51).

### Deglycosylation using PNGase F and SDS-PAGE.

Ten microliters of the sucrose-gradient-purified viruses (virus concentration of 10 μM, which was determined using an enzyme-linked immunosorbent assay against the virus NP as described previously [28]) was treated with PNGase F enzyme at 37°C for 24 h according to the manufacturer’s instructions (New England BioLabs). The viruses were then mixed with NuPAGE LDS sample buffer (4×) and heated at 70°C for 10 min before being loaded onto NuPAGE 4 to 12% bis-Tris protein gels for SDS-PAGE analysis (Life Technologies). The viral protein bands in the gel were revealed by staining with Pierce PageBlue protein staining solution (Thermo Scientific).

### Statistical analysis.

Statistical analysis was performed using GraphPad Prism 8 (GraphPad Software). One-way analysis of variance (ANOVA) and two-way ANOVA were used to test differences between different groups. The two-way ANOVA (Tukey's multiple-comparison test) results for Fig. 3A and B are in Table 1 and 2; *P* values of <0.05 were considered significant.
